# Mindfulness as a well-being initiative for future nurses: a survey with undergraduate nursing students

**DOI:** 10.1186/s12912-021-00783-0

**Published:** 2021-12-20

**Authors:** Clare Mc Veigh, Joanne Reid, Claire Carswell, Lindsay Ace, Ian Walsh, Lisa Graham-Wisener, Soham Rej, Angela Potes, Karen Atkinson, Trudi Edginton, Helen Noble

**Affiliations:** 1 School of Nursing and Midwifery, Queen’s University Belfast, Belfast, UK; 2 School of Medicine, Dentistry and Biomedical Sciences, Queen’s University Belfast, Belfast, UK; 3 Centre for Improving Health-Related Quality of Life, School of Psychology, Queen’s University Belfast, Belfast, UK; 4 McGill Meditation and Mind-Body Medicine Research Clinic (MMMM-RC) and Geri-PARTy Research Group, Jewish General Hospital, Montreal, Canada; 5 Department of Psychiatry, McGill University, Montreal, Canada; 6 Mindfulness UK, Taunton, Somerset UK; 7 School of Arts and Social Sciences, Department of Psychology, City University of London, London, UK

**Keywords:** Indfulness, Nursing students, Undergraduate, Healthcare, Meditation

## Abstract

**Background:**

Mindfulness can potentially positively impact well-being and resilience in undergraduate nursing students. The psychological well-being of such students undertaking clinical training is paramount to ensure optimal learning, and to equip them with skills to manage their wellbeing in future clinical practice. The aim of our study was to explore the views of undergraduate nursing students in relation to understanding and engaging with mindfulness, and how mindfulness could best be delivered within their university programme.

**Methods:**

An online survey was administered via a cloud-based student response system to a convenience sample of first year undergraduate nursing students completing a Bachelor of Science (BSc) Honours (Hons) degree in nursing at a University in the United Kingdom. Data were analysed using descriptive statistics and thematic analysis.

**Results:**

The survey achieved a response rate of 78% (*n* = 208). Seventy-nine percent of participants had heard of mindfulness and were interested in taking part in a mindfulness programme. Respondents reported that the ideal delivery of the programme would consist of weekly 45-min, in person group sessions, over a 6-week period. Respondents also indicated that a mobile application could potentially facilitate participation in the programme. Thematic analysis of open-ended comments, and free text, within the survey indicated 4 overarching themes: 1) Perceptions of what mindfulness is; 2) Previous mindfulness practice experiences; 3) Impact of mindfulness in nursing; 4) The need for a future well-being initiative for undergraduate nursing students.

**Conclusions:**

Undergraduate nursing students perceived that a mindfulness programme has the potential to enhance well-being and future clinical practice. This student cohort are familiar with mindfulness and want more integrated within their undergraduate curriculum. Further research is required to examine the effectiveness of a tailored mindfulness intervention for this population that incorporates the use of both face-to-face and mobile delivery.

## Background

Psychological morbidity is recognised as a growing global concern amongst nursing students [1, 2]. Evidence suggests that the nature of healthcare education can contribute to the high levels of psychological morbidity amongst health service students [3–5], with clinical placements being identified as a particular stress trigger [6]. It is internationally recognised that engaging in mindfulness can positively impact the holistic wellbeing and resilience of nursing and medical students [7–9]. Enhanced psychological well-being of students undertaking clinical training is essential to ensure optimal learning, and also to prepare them for future healthcare challenges [10]. Mindfulness has the potential to fit well within a continuum of best practice in healthcare [11].

Mindfulness has been described as being able to have present-moment awareness, in a non-judgemental manner and without criticism [12]. Mindfulness may be obtained through purposively focusing fully on present experiences [13], thus providing a simplistic platform by which participants can enhance their own well-being [14]. However, formal mindful practices (e.g. breath awareness, body scans) can enhance the capacity for present-moment awareness and acceptance/non-judgment [15]. When utilising mindfulness, improvements may be experienced in relation to attention [16, 17], well-being [18, 19] and resilience [20, 21] in both clinical and non-clinical populations. Resilience is a trait that can help people cope with adversity, trauma and difficult events. It is an ineffable quality which allows individuals to cope effectively with failure; often supported by a positive and optimistic attitude. Within the healthcare profession, resilience plays a key role in enhancing the quality of care delivered to patients and workforce sustainability [22]. In India, a cross sectional survey of undergraduate medical students (*n* = 353) reported that psychological symptoms such as depression (51.3%), anxiety (66.9%) and stress (53%) were experienced by over half of participants [23].

Participating in mindfulness interventions can increase well-being amongst nursing students [9] by reducing symptoms such as anxiety and stress. It has previously been highlighted that nurses can experience higher levels of stress-related burnout, in comparison with other health care professionals [24]. Burnout within the workplace can have a negative impact on the bio-psychosocial well-being of nursing staff, as well as impacting patient care, and career satisfaction [25]. Additionally, stress and burnout can lead to higher staff turnover amongst registered nurses [26] and newly qualified nurses [27]. An Australian study exploring the implementation of a 7-week mindfulness program, delivered using an audio CD, for nursing and midwifery students reported an increase in general well-being, and enhanced clarity of thought amongst this population [28]. In China, a face-to-face mindfulness intervention had a positive impact on nursing students’ physiological health through lowering blood pressure [29]. Amongst medical students, mindfulness not only decreased perceived levels of stress but also increased self-compassion [30]. However, due to academic and clinical demands nursing students may not avail of traditional face-to-face mindfulness interventions [14].

Alongside the growing recognition of mindfulness practices, there is an increasing awareness that mindful meditation interventions need to be more accessible to participants [31]. One way in which mindfulness programs can be more readily accessed is through the use of online technology. Amongst newly qualified registered nurses, previous research highlighted that a mindfulness intervention delivered online reduced anxiety [32]. However, the most effective delivery of mindfulness interventions in helping to promote well-being and resilience amongst nursing students is unclear. The aim of our study was to identify the views of undergraduate nursing students in relation to mindfulness, and how it could be embedded within their university curriculum.

## Method

### Design

A cross-sectional online survey was administered via a cloud-based student response system that students could access via their computer or handheld mobile device, to identify participants’ perceptions and experiences in relation to mindfulness meditation and its implementation.

### Setting

This survey involved first year undergraduate nursing students enrolled on the BSc (Hons) Nursing programme at a University in Northern Ireland. A mindfulness initiative is not currently available to this student cohort.

### Sample and participants

A convenience sample of undergraduate nursing students completing a BSc Hons Degree in Nursing were approached. All participants were in the first year of the nursing programme and had completed two clinical placements. This sample was chosen as students had both experience of clinical placement and the university setting, and the sample was readily accessible to the researcher [33]. Participants were provided with details about the study prior to the survey being administered. Consent to participate in the study was implied by completion and submission of the survey via the cloud-based platform.

### Ethical considerations

The study received ethics approval from the Queen’s University Belfast, School of Nursing and Midwifery Research and Ethics Committee (Ref: C.McVeigh.05.19.M1.V1).

### Instrument

The 21-item survey (Table 1) was developed by the authors based on previous pilot research [8], and a review of the literature [1–11, 28, 29]. It consisted of demographic questions (*n* = 3), multiple-choice items (*n* = 8) and open-ended questions (*n* = 5). Questions focused on participants understanding of mindfulness, their past and present engagement with mindfulness practice, and their views on how a mindfulness programme could be integrated into the undergraduate nursing curriculum.

**Table 1:** **Survey**

1. Have you heard of mindfulness? YES/ NO *If No, please go directly to question 8.*	
2. What is your understanding of Mindfulness?	
3. Have you engaged in mindfulness practice? YES/ NO	
4. If you answered YES to question 3, please describe the mindfulness practice you engaged in.	
5. If you have engaged in mindfulness practice, did it help you and how?	
6. Do you practice any other meditation/ mind-body practices (e.g. yoga, tai chi)? YES/ NO	
7. If you answered YES to question 6, how often do you practice each week?	
8. Would you be interested in taking part in a new mindfulness programme with other nursing students? YES/ NO	
9. How long do you think a weekly group mindfulness session should be? A. 45 min B. 60 min C. 90 min D. 2 h E. Other	
10. If you chose OTHER, please explain.	
11. How many weeks long should the mindfulness programme be? A. 4 weeks B. 5 weeks C. 6 weeks D. 8 weeks E. Other	
12. If you chose OTHER, please explain.	
13. In addition to the group mindfulness programme, would you be happy to do personal mindfulness practice in your own time? YES/NO	
14. Do you think a student well-being initiative should be part of the nursing curriculum?	
15. How do you think mindfulness practice may benefit you as a nurse in clinical practice?	
16. Are there any barriers that might prevent you from participating in mindfulness practice?	
17. Which of the following do you think may help to facilitate your participation in mindfulness practice? PLEASE SELECT ALL THAT APPLY A. Student champion B. A Mobile APP C. Student-led lunchtime meditation sessions D. Other	
18. If you chose OTHER please explain.	
19. What is your age group? A. 18–22 years B. 23–30 years C. 31–40 years D. 41–50 years E. 50+ years	
20. What is your gender? A. Male B. Female C. Other	
21. What is your Ethnic Group? A. White British B. White Irish C. Irish Traveller D. Any other White Background E. White and Black Caribbean F. White and Black African G. White and Asian H. Any other Mixed/ Multiple Ethnic Background I. Indian J. Pakistani K. Bangladeshi L. Chinese M. Any other Asian Background N. African O. Caribbean P. Any other Black Background	

### Data collection

The questionnaire took approximately 10 to 15 min to administer and complete. Participants were approached at the beginning of a lecture and asked to participate anonymously using the cloud-based student response system that they had been using previously in class. The study was conducted in May 2019.

### Analysis

Closed response survey data were entered into SPSS version 24 [34] and descriptive statistics were used to summarise the data. Free text responses were analysed by the lead author (CMV) using thematic analysis [35] to identify overarching themes that emerged from the data. Themes were then reviewed by other members of the research team (JR, CC, IW, LGW, SR, AP, KA, TE, HN). Stage one of analysis involved assigning descriptive themes to sections of the data, whilst stage two encompassed grouping together these descriptive themes to generate interpretative themes that highlighted emerging patterns within the data [36]. The final stage identified a number of overarching themes that were developed by pulling together and linking all the interpretative themes.

## Results

Two hundred and sixty-six students were approached to participate in the study, and from those 208 consented giving a response rate of 78%. Demographic details are presented in Table 2. Amongst respondents, the majority were between the ages of 18–22 years (51.9%), female (84.6%) and white Irish or British (83.6%). Closed response survey data is presented in Table 3.

**Table 2:** Demographic characteristics

Sex	*N* (%)
***Female***	176 (84.6%)
***Male***	10 (4.8%)
***Other***	0
***Question not answered by participants***	22 (10.6%)
Age	
***18–22 years***	108 (51.9%)
***23–30 years***	45 (21.6%)
***31–40 years***	30 (14.4%)
***41–50 years***	3 (1.5%)
***50 years +***	0
***Question not answered by participants***	22 (10.6%)
Ethnic Group	
***White Irish or British***	174 (83.6%)
***African***	1 (0.5%)
***Any other Asian background***	1 (0.5%)
***Any other Mixed/Multiple ethnic background***	2 (1.%)
***Any other White background***	4 (1.9%)
***White and Black African***	1 (0.5%)
***Question not answered by participants***	25 (12%)

**Table 3:** Closed response survey data

Question	***N*** (%)
***Have you heard of mindfulness?***	
***Yes***	159 (76.4%)
***No***	43 (20.7%)
***Not answered***	**6 (2.9%)**
***Have you engaged in mindfulness practice?***	
***Yes***	77 (37%)
***No***	84 (40.4%)
***Not answered***	47 (22.6%)
***Do you practice any other meditation/ mind-body practices (*e.g.** ***yoga, tai chi)?***	
***Yes***	37 (17.8%)
***No***	157 (75.5%)
***Not answered***	14 (6.7%)
***Would you be interested in taking part in a new mindfulness programme with other nursing students?***	
***Yes***	141 (67.8%)
***No***	55 (26.4%)
***Not answered***	12 (5.8%)
***How long do you think a weekly group mindfulness session should be?***	
***45 min***	116 (55.8%)
***60 min***	47 (22.6%)
***90 min***	2 (1.%)
***2 h***	1 (0.5%)
***Other***	14 (6.7%)
***Not answered***	28 (13.4%)
***How many weeks long should the mindfulness programme be?***	
4 weeks (36.7%)	66 (31.8%)
5 weeks (4.4%)	8 (3.9%)
6 weeks (45%)	81 (38.9%)
8 weeks (6.1%)	11 (5.3%)
Other (7.8%)	14 (6.6%)
Not answered	28 (13.5)
***In addition to the group mindfulness programme, would you be happy to do personal mindfulness practice in your own time?***	
***Yes***	162 (77.9%)
***No***	30 (14.4%)
***Not answered***	16 (7.7%)
***Which of the following do you think may help to facilitate your participation in mindfulness practice?***	
***Please select all that apply.***	
***Student Champion***	27 (13%)
***A Mobile APP***	149 (71.6%)
***Student Led Lunchtime Meditation Sessions***	43 (20.7%)
***Other***	14 (6.7%)
***Not answered***	26 (12.5%)

The majority of respondents had heard of mindfulness (76.4%), and 37% had engaged in a mindfulness practice. Additionally, 17.8% of respondents reported that they had engaged in other meditation/mind-body practices. The majority of respondents (67.8%) reported that they would be interested in taking part in a mindfulness programme with other nursing students. A chi-square statistic was calculated to examine any association between participants who had heard of mindfulness and those who would be interested in taking part in a new programme, but there was no significant association (*p* > 0.05). When asked about the preferred length of a weekly mindfulness sessions the majority of respondents (55.8%)reported 45 min as their preferred length of time. With regards to the length of the mindfulness programme itself, the majority of students (38.9%) indicated that 6 weeks was the ideal length of time in which to deliver the full programme. In addition, over three quarters of nursing students (77.9%) reported that they would be happy to engage in personal mindfulness practice in their own time, alongside the mindfulness programme. This was not significantly associated (*p* = 0.476) with whether these nursing students had heard of mindfulness before. Interestingly, 71.6% of respondents indicated that a mobile application could potentially help facilitate their participation in a mindfulness programme. Others indicated that lunchtime meditation sessions (20.7%) or a student champion (13%) may help enhance participation in the programme.

Thematic analysis of the open-ended responses generated 4 overarching themes (Table 4): *Perceptions of what mindfulness is*; *Previous mindfulness practice experiences*; *Impact of mindfulness in nursing*; *The need for a future well-being initiative for undergraduate nursing students*. The first overarching theme, *perceptions of what mindfulness is,* highlighted participants views of mindfulness as a strategy to promote self-care that could also enable them in being more mindful of others. Additionally, mindfulness was described as related to enhancing awareness of the present moment and of their thoughts. The overarching theme; *previous mindfulness practice experiences,* pertained to participants’ perceptions of their previous mindfulness practice. Many participants felt it had helped them to relax and enhanced their mental well-being. However, some participants additionally indicated that previous mindfulness practice had not been beneficial to them due to factors such as; not practicing long enough to benefit from mindfulness, perceiving mindfulness to have increased their stress or finding the practice of no benefit. Overarching theme 3, *impact of mindfulness in nursing,* described how mindfulness could potentially benefit students undertaking the undergraduate nursing programme. It was also recognised that there might be barriers to student participation. Participants additionally highlighted that mindfulness had the potential to positively impact their future clinical practice. Within *the need for a future well-being initiative for undergraduate nursing students* (overarching theme 4), responses conveyed the perception amongst participants that due to the stressful nature of their nursing programme, a well-being initiative may be beneficial for students and aid in the student not being ‘*forgotten.’* Perceptions highlighted that a future initiative should help enhance the well-being of students so they can optimally care for others. However, several participants conveyed the importance of future well-being initiatives as optional for nursing students and not a compulsory element of the curriculum.

**Table 4:** Thematic analysis of open-ended questions and free text comments

Overarching Themes	Interpretative Themes	Participant quotes
Perceptions of what mindfulness is	Mindfulness is about self-care	*Mindfulness is about inner peace and looking after your mental health*
		*Mindfulness is a way of unwinding and going to a better place*
		*Mindfulness is to reduce stress*
	Being mindful of others	*You have to look after yourself to look after others*
		*I think mindfulness means to be open without judgement. To be empathetic towards others and how your words and actions can help or hinder others*
	Enhanced awareness	*Mindfulness is being aware of your thoughts and feelings in response to external influencing factors*
		*Being aware of what is going on at the present moment, being present and not hooked up in the past or future*
		*Taking time to be at peace with your thoughts and emotions*
Previous Mindfulness Practice experiences	Helped to relax	*Mindfulness helped to relax my mind and provide peace*
		*I loved meditation as it helped me focus on that moment on my breathing and sensations throughout my body, so I was distracted from everything else*
		*Mindfulness does help before I go to sleep*
	Enhanced mental well-being	*Mindfulness alleviated stress and made me happier*
		*Mindfulness removed some worrisome thoughts from my head, validated my feelings and allowed me time to focus on more important things*
		*Mindfulness helped me to stop being so anxious and to stop worrying about the future so much*
	Previous mindfulness practice not beneficial	*I didn’t stick at mindfulness long enough to see any sort of development*
		*Mindfulness didn’t help, it made me more stressed and I just didn’t get it*
		*I found mindfulness a waste of time*
Impact of mindfulness in nursing	Benefits to students undertaking the undergraduate nursing programme	*Help you to keep the stress of the nursing course and profession overall down, as it is a demanding full-time course, and if you don’t practice mindfulness* etc. *your mental health could become worse due to these stresses*
		*Mindfulness would be a good way to relieve stress and understand the thoughts that are going through your head as well as better equip students for life after university*
	Benefits to future clinical practice	*Mindfulness will help me appreciate my skills and assure me that I am a good nurse*
		*Mindfulness would allow me to recognise the signs of burnout and mental fatigue, both in myself and other people, and give me the right tools to help combat negative feelings*
		*Mindfulness would help me to cope with the pressure of clinical practice and increase compassion and communication skills*
	Barriers to mindfulness in undergraduate nursing programme	*I don’t personally believe in mindfulness*
		*The previous time I tried a mindfulness class actually stressed me out more as doing nothing made me feel lazy*
		*Timing is a barrier of course. For example, if it would interfere with study for exams or completing assignments*
		*Time constraints and stress*
The need for a future well-being initiative for undergraduate nursing students	Nursing course is stressful	*This course is very demanding and experiences on placement can have a major impact on nursing students*
		*The pressure and workload of the course can become overwhelming, especially around the end of the year. A well-being initiative would be useful to help student nurses cope with stress and learn how to balance university, placement and home commitments.*
	Student forgotten	*The course is often focussed solely on the patient therefore the professional, or even the student, I personally feel are not regarded.*
		*Sometimes the student’s well-being can be forgotten about, it’s important that they are well in order to attend university and complete certain tasks given to them.*
	Take care of the students so they can take care of others	*Taking care of our [the student] health and wellbeing first and foremost so we can help others*
		*A well-being initiative would help teach future self-care methods for our nursing career*
	Future well-being initiative should not be mandatory	*A future well-being initiative should be available but maybe not mandatory*
		*A well-being initiative could benefit some students, but it may not because missing a session can lead to more stress. The curriculum is already full time and it would be difficult to add more to it.*

## Discussion

This study produced new knowledge in relation to undergraduate nursing students’ perceptions of a mindfulness- based well-being initiative within the university curriculum. Previous research has indicated a lack of standardised approach to delivering a mindfulness programme to undergraduate nursing students [37]. Within the present study, participants indicated that the ideal delivery for a mindfulness programme would incorporate weekly 45—minute face-to-face sessions over a 6-week period. Participants in the present study expressed willingness to engage in their own mindfulness practice, alongside the mindfulness programme.

An additional finding of the present study indicated that the majority of undergraduate nursing students would prefer a mobile application to aid their participation in mindfulness meditation. Previous research exploring mindfulness practice delivered by a mobile application to medical students, highlighted decreased perceived stress and increased general well-being amongst participants [38].

A 12-week Mindfulness Based Stress Reduction (MBSR) programme for Turkish nursing students found increased awareness of staying in the present amongst participants [39]. Nursing students who participated in the present study perceived that mindfulness would enable them to enhance their awareness, but also encourage self-care. Data additionally demonstrated the potential impact of mindfulness on nursing practice at undergraduate level. Due to the demanding nature of their course, nursing students believed mindfulness could potentially help build coping strategies to deal with stress. Those who had engaged in previous practice conveyed that it had aided relaxation and enhanced their mental well-being through alleviating stress or anxiety. Previous research has similarly demonstrated perceived reduction in stress and anxiety amongst nursing students who engaged in MBSR [9, 32, 40, 41]. Engaging in mindfulness practice has also been shown to improve quality of life amongst both undergraduate nursing students, and registered nurses [42].

The benefits of mindfulness have not only been recognised amongst undergraduate nursing students, but also registered nurses working in clinical settings. Previous research involving newly registered nurses (*n* = 5) in Canada investigated the effectiveness of a shortened version of the 8-week Mindfulness Based Stress Reduction (MBSR) course [43]. The shortened MBSR programme aided participants in recognising their stress levels, increasing their self-compassion and recognising their capabilities and limitations in clinical practice. Similarly. within the present study participants expressed that participating in a mindfulness programme might aid them in recognising personal signs of burnout and mental fatigue which could enhance coping strategies when practicing as a registered nurse. In addition, participants felt that mindfulness practice could increase compassion and improve clinical practice if they were aware of how well they were doing their job. Previous pilot research by the current authors [8] found that a 6-week mindfulness course could increase mindfulness and resilience amongst both nursing and medical students. Mindfulness has the potential to enhance resilience amongst future nurses working in clinical practice.

Within the present study some participants who had previously engaged in mindfulness practice felt it was unhelpful and even exacerbated their stress. Some additionally expressed that a mindfulness programme would not be feasible within the current undergraduate nursing curriculum due to time constraints and the busy nature of the programme. Previous research with trainee social workers recommended that mindfulness may not appeal to all students and therefore should not be mandatory [43]. Another study highlighted that a 7-week MBSR course delivered as a compulsory element to first year undergraduate medical students, had lower levels of satisfaction and feedback compared to an optional course for 2nd year medical students [44]. The present study additionally highlighted that this is the perception in relation to well-being initiatives in general, and not just mindfulness-based interventions. However, the majority of UG nursing students recognised the need for a non-compulsory well-being strategy within the undergraduate nursing curriculum, for clinical practice and promote self-care.

### Limitations and future research

This study included a convenience sample of participants from one university and therefore results may not be generalisable. In addition, the survey instrument was developed specifically for this study with no psychometric testing. However, the survey was designed by the authors based on previous pilot research and a review of the literature. With regard to future research, an extension of this study is recommended adopting the Medical Research Council (MRC) framework for complex interventions [45] to develop a mindfulness meditation programme for UG nursing students. This would initially involve conducting a feasibility study with undergraduate nursing students to explore the practicalities of implementing the proposed programme. This would be followed by an interventional study to determine the effectiveness of the programme on a number of outcomes This stage would focus on testing the intervention based on the data obtained from the feasibility study.

## Conclusions

In conclusion, findings from the survey suggested that implementation of a mindfulness meditation programme would be acceptable to undergraduate nursing students. The results of this study provide important insights regarding how this programme may be optimally delivered using face-to-face sessions, and a mobile platform. The importance of ensuring that a well-being initiative is not a mandatory element of the undergraduate nursing curriculum has also been highlighted. Further research is needed to explore the feasibility of implementing a blended approach mindfulness programme for undergraduate nursing students.

## Data Availability

The datasets used and/or analysed during the current study are available from the corresponding author on reasonable request. The data set generated during the study will not be shared in order to respect confidentiality and not to compromise research participant privacy.

## Abbreviations

**BSc**: Bachelor of Science
**Hons**: Honours
**MBSR**: Mindfulness-Based Stress Reduction
